# Organelle Genome Characteristics and Phylogenetic Analysis of a Warm-Season Turfgrass *Eremochloa ophiuroides* (Poaceae)

**DOI:** 10.3390/biology14080975

**Published:** 2025-08-01

**Authors:** Junming Zhao, Yanli Xiong, Maotao Xu, Wenlong Gou, Tingyong Yang, Yi Xiong, Zhixiao Dong, Ling Pan, Lina Sha, Hong Luo, Xiao Ma

**Affiliations:** 1 College of Grassland Science and Technology, Sichuan Agricultural University, Chengdu 611130, China; junmingzhao163@163.com (J.Z.); yjstgsyxmt@163.com (M.X.); xiongyi95@126.com (Y.X.); dongzhixiao94@126.com (Z.D.); shalina@sicau.edu.cn (L.S.); 2 Sichuan Academy of Grassland Sciences, Chengdu 611731, China; yanlimaster@126.com; 3 School of Life Science and Engineering, Southwest University of Science and Technology, Mianyang 621010, China; gsz080115@163.com; 4 Ganzi Prefecture Grassland Workstation, Ganzi Tibetan Autonomous Prefecture, Kangding 626000, China; 18990480460@163.com; 5 College of Animal science and Technology, Yangzhou University, Yangzhou 225009, China; panling1199@126.com; 6 Department of Genetics and Biochemistry, Clemson University, Clemson, SC 29634, USA

**Keywords:** centipedegrass, organelle genome, gene transfer events, mitochondrial marker

## Abstract

Plant mitochondrial genomes are characterized by their complex compositions and structures, large genomes, rapid recombination and evolution rates, and frequent intracellular gene transfer events. Centipedegrass, known as “Chinese turfgrass”, is a warm-season turfgrass that exhibits excellent tolerance to both biotic and abiotic stresses. The chloroplast genome, with 139,107 bp, and the mitochondrial genome, with 564,432 bp, were both assembled into a single circular structure. We identified 44 gene transfer events between the chloroplast and mitochondrial genomes. The mitochondrial gene *cox1* could serve as a marker for distinguishing accessions found at different altitudes. The unique features of the centipedegrass mitochondrial genome, coupled with the comparative genomic analysis of both chloroplast and mitochondrial genomes, have the potential to enrich the Poaceae database and provide crucial perspectives on plant evolution, energy metabolism, and responses to environmental conditions. The markers developed could facilitate the analysis of the genetic diversity of centipedegrass.

## Simple Summary

The Poaceae family plays a pivotal role in both human sustenance and environmental ecosystems, encompassing staple crops such as wheat, rice, and corn that form the cornerstone of global food security. Despite the agricultural and ecological importance of Poaceae species, mitochondrial genomes have been sequenced for merely 23 species within this family, hindering comprehensive investigations of its phylogenetic structure, evolutionary history, and genetic diversity across Poaceae. In this study, the complete organelle genomes of a warm-season turfgrass, centipedegrass, were reported, representing the first mitogenome for the *Eremochloa* genus within Poaceae. These genomes provide a crucial genomic resource for future assembly and comparative studies of related species. Our analysis revealed repetitive sequences and evidence of intracellular gene transfer (IGT) events between chloroplast and mitochondrial genomes. Furthermore, in this investigation, organelle-based molecular markers were developed, and the mitochondrial cox1 gene was identified as a potential diagnostic marker for distinguishing accessions adapted to different altitudinal ranges.

## 1. Introduction

Centipedegrass [(*Eremochloa ophiuroides* (Munro) Hack.] is the only species among the eight species in the *Eremochloa* genus (Poaceae family) that can be used as turfgrass. Known for its tolerance to drought, pests, and diseases, as well as its low maintenance requirements, centipedegrass has been recognized as a world-renowned warm-season turf grass and an excellent material for green construction [1]. In addition to its limited distribution in Southeast Asia, it is predominantly found in the Yangtze River basin and the southern part of China, where it is commonly referred to as “Chinese turfgrass”. It is extensively utilized in public green spaces and lawn construction for soil and water conservation purposes [2]. Additionally, centipedegrass has traditional medicinal applications, including the treatment of abdominal pain following heat stroke [3]. With the global scarcity of water and energy resources and heightened awareness regarding environmental issues, the selection and breeding of turfgrass varieties that possess a moderate texture, high adaptability, and low maintenance requirements have become a pressing challenge [1]. Owing to its distinctive morphology, growth characteristics, and management practices, centipedegrass has garnered increased attention and is widely employed in turf production and environmental greening projects. However, although China is the species’ center of origin and possesses abundant genetic resources, the absence of comprehensive genetic diversity assessments precludes a full understanding of centipedegrass’s resistance to environmental changes. This may lead to valuable genetic resources potentially being overlooked. Furthermore, the lack of detailed research on environmental adaptation and critical biological processes, such as energy metabolism and respiration, represents another barrier to the breeding and practical application of this species.

Chloroplasts and mitochondria are the sole organelles in plants with genomes of endosymbiotic origin, serving as the central sites for photosynthesis and respiration, respectively [4]. Due to the abundance of repeats and fragments resulting from intracellular horizontal transfer within plant mitochondrial genomes, the assembly of all contigs into a single master chromosome presents a significant challenge. This has led to an uneven distribution of genome sequencing efforts across different organelles and taxa [5]. To date, the National Center for Biotechnology Information (NCBI) has documented a total of 1100 organelle genomes of Poaceae (https://www.ncbi.nlm.nih.gov/datasets/organelle/, accessed on 30 May 2025), of which only 23 are mitochondrial genomes. Plant mitochondrial genome sequences evolve at a slower rate, making them particularly suitable for phylogenetic studies at early plant stages and on a large scale [6]. Moreover, the analysis of complete mitochondrial genomes provides more taxonomic resolution compared to that of chloroplast genomes [7]. However, the contribution of the entire mitogenome to phylogeographic or taxonomic research within the same genus is not uniform, and some regions that appear homologous may not be true orthologs [7]. Therefore, the construction of mtDNA phylogenies at the individual level should ideally be based on either the complete pan-mitogenome or conserved core fragments present in all mitogenomes, with a focus on regions containing functional genes [8,9,10]. For example, the mitochondrial cytochrome c oxidase subunit I (*cox1*) has been utilized as an identification system for different animal groups [11] and as a molecular marker in higher plants [12].

In this study, we present the first complete assembly and characterization of the centipedegrass mitochondrial genome, representing a pioneering contribution to the organelle genomics of this genus. Our study aims to: (1) perform a complete functional annotation of centipedegrass organelle genomes, (2) conduct comprehensive codon usage bias analysis across all coding sequences, (3) identify and characterize repetitive genomic elements, (4) investigate intracellular gene transfer events, and (5) develop and validate novel organelle-specific molecular markers for breeding applications. These genomic resources significantly expand the current understanding of centipedegrass biology while providing valuable tools for its genetic improvement and breeding applications.

## 2. Materials and Methods

### 2.1. Sample Collection and Sequencing

Healthy and young leaves were harvested and employed for the isolation of total DNA utilizing the CTAB method. DNAs that met the quality criteria were subjected to random fragmentation, followed by damage repair and A-tail preparation, prior to library construction. Short-read sequencing libraries were constructed with the Illumina TruSeq DNA kit (Illumina, Inc., San Diego, CA, USA) and sequenced on a HiSeq X system (Macrogen Inc., Seoul, Republic of Korea) to produce 150 bp paired-end reads with 350 bp insert sizes. To obtain a high-quality, complete mitogenome assembly of centipedegrass, we employed PacBio HiFi circular consensus sequencing. Genomic DNA was processed using the SMRTbell Express Template Prep Kit 2.0 (Pacific Biosciences, Menlo Park, CA, USA) and sequenced on a PacBio Sequel II system (Pacific Biosciences, Menlo Park, CA, USA), yielding HiFi reads with 15–20 kb average length and >99% single-molecule accuracy.

### 2.2. Organelle Genome Assembly and Annotation

The clean data, processed through filtering with fastp v 0.23.4 [13] with the parameters ‘–q 20 | -l 50 | -w 4’, were used for the assembly of the chloroplast genome. To simplify the assembly process, the clean reads were aligned against the chloroplast genome database created by Genepioneer using the ‘very-sensitive-local’ model implemented in bowtie2 v2.5.4 [14]. The aligned reads were then used for de novo assembly of the chloroplast genome using SPAdes v4.2.0 software, with kmer settings of 55, 87, and 121 [15]. For mRNA, rRNA, and tRNA annotation, the following tools were used: prodigal v2.6.3 [16], hmmer v3.4 [17], and aragorn v1.2.36.c [18].

The raw sequencing reads were subsequently filtered using the filtlong v0.2.1 software with ‘--min_length 1000 | --min_mean_q 7’ options. The filtered reads were then aligned to the mitochondrial core gene database (https://github.com/xul962464/plant_mt_ref_gene, accessed on 10 June 2025) using minimap2 v2.30 [19] to obtain seed sequences. Next, next-generation sequencing (NGS) data were aligned with the polished third-generation data, which were processed with canu v.2.2 [20]. The aligned NGS data and the polished third-generation data were merged using Unicycler v0.5.1 [21] with ‘--mode normal, --keep 3’ options. The resulting assembly was visualized and manually adjusted with Bandage v0.9.0 [22] to ensure continuity and greater than 90% coverage/depth. Protein-coding genes (PCGs) and rRNA were annotated by aligning the sequence to published plant mitochondrial annotation data using blast v2.10.1+ [23], while tRNAs were annotated with tRNAscanSE 2.0 [24].

### 2.3. Codon Usage Bias Analysis

The relative synonymous codon usage (RSCU) values for each codon were calculated using CAI v1.0.2. To analyze the factors contributing to codon bias, excluding base composition, the effective number of codons (ENC) and the occurrence frequency of guanine and cytosine at the 3rd synonymous codon (GC3s) were computed using codonW v2.7.2.1. Additionally, the relationship between ENC and GC3s was visualized through the creation of simulation curves, which were plotted using the ggplot package in R.

### 2.4. Repeat Sequences Identification

Three types of repeats, including simple sequence repeats (SSRs), tandem repeats, and dispersed repeats, were identified within the organelle genomes using misa v2.1 [25], trf v4.09.1 [26], and blastn v2.14.1+ software [23] with the parameters ‘1-10 | 2-6 | 3-5 | 4-5 | 5-5 | 6-5’, ‘2 7 7 80 10 50 500’, and ‘-e 1e-5’, respectively. The repeat sequences within the mitochondrial genome were then visualized using the circus tool [27] to provide a comprehensive graphic representation of these repetitive elements.

### 2.5. Gene Transfer Events Between Chloroplast and Mitochondrial Genomes

Homologous sequences between the chloroplast and mitochondrial genomes were used to identify gene transfer events. By using blast v2.10.1+ software with an E-value of 1 × 10^−5^, the chloroplast genome sequences were aligned to the mitochondrial genome. The resulting alignments were subsequently visualized using circus v0.17.1 [27].

### 2.6. Phylogenetic Analyses in the Gramineae Family

The shared genes of the chloroplast and mitochondrial genomes of 24 Gramineae species were isolated and aligned with Clustalw v2.1 [28]. The Neighbor-Joining (NJ) trees based on chloroplast and mitochondrial genes were constructed using iqtree v1.6.12 [29] and visualized in Figtree v1.4.4. To compare these two trees, a tangled tree was constructed using the tanglegram function. The divergence time of the 24 Gramineae species was estimated with the BEAST v1.7.0 package [30], with the substitution model of HKY. The tree prior was determined as the Yule process with the fossils of *Triticum aestivum* and *Aegilops speltoides* (1.72 Mya) in the mitochondrial tree, and *Triticum timopheevii* and *Aegilops speltoides* (1.30 Mya) in the chloroplast tree, respectively. Each MCMC run had a chain length of 100,000,000 with sampling every 10,000 steps. Then the divergence time was estimated with the Treeannotator v1.10 program [30].

### 2.7. Structural Variation Among Mitochondrial Genomes

To investigate the structural variation (SV) and collinearity among mitochondrial genomes, centipedegrass and 11 closely related species, determined via phylogenetic analysis, were used. The pairwise alignment was performed using minimap2 v2.30 [19] with the default parameters, and then the SyRI v1.7.1 pipeline [31] was used to identify sequence and structural variations. These variants were annotated using snpEff v5.2f [32] and visualized using plostr v1.1.0 [33].

### 2.8. Organelle Genome Marker Development and Utilization

Two mitochondrial genes (*cox1* and *atp6*) and two intergenic regions (*psbA-trnH* and *rps-tRNAS*) (Table S1) were chosen for amplification and sequencing. They were considered suitable polymorphic organelle genome markers for genetic diversity studies. DNAs from a total of 24 wild centipedegrass germplasms (Table S2), collected from Chongqing (China), Sichuan (China), and Guizhou (China), were used for PCR amplification of the targets following the protocol outlined by Xiong et al. [34]. The PCR products were subsequently purified and recovered using the Universal DNA Purification Kit (Tiangen Co., Ltd., Beijing, China), and the purified DNA was employed for Sanger sequencing. Sequencing artifacts were minimized by removing the first and last 100 bp of sequence from each marker before subsequent analysis. The sequences of each marker were submitted to BLAST v2.10.1+ [23] for comparison, and genetic diversity parameters were calculated using DnaSP 6 software [35]. The NJ phylogenetic trees were constructed based on these markers using MEGA 4 [36].

## 3. Results

### 3.1. The Assembly and Annotation of the Organelle Genomes of Centipedegrass

**Figure 1:**
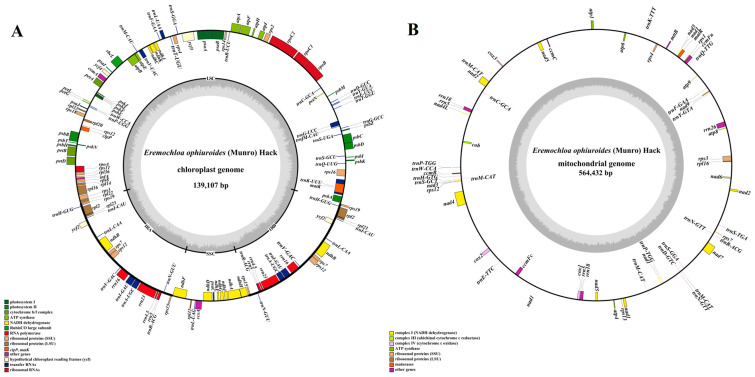
The genome maps of the chloroplast (**A**) and mitochondrial genomes (**B**) of centipedegrass. In the circular gene map, the transcription direction is indicated by arrow orientation: genes on the outer circle are transcribed clockwise, while inner circle genes are transcribed counterclockwise. The GC content is represented by dark gray shading, with light gray indicating the AT content. LSC, large single-copy region; IR, inverted repeat; SSC, small single-copy region.

Combining second-generation (depths of 1150X) and third-generation sequencing (366X), the chloroplast and mitochondrial genomes of centipedegrass were successfully assembled into single circular structures (Figure 1), with a total length of 139,107 bp and 564,432 bp, respectively. The chloroplast genome contains a total of 132 genes, comprising 86 protein-coding genes, 38 tRNA genes, and 8 rRNA genes (Figure 1A, Table S3), while the mitochondrial genome contains 63 genes, including 37 protein-coding genes, 21 tRNA genes, and 5 rRNA genes (Figure 1B, Table S3). Notably, no pseudogenes were identified within the organelle genomes of centipedegrass.

### 3.2. Codon Bias Analysis

**Figure 2:**
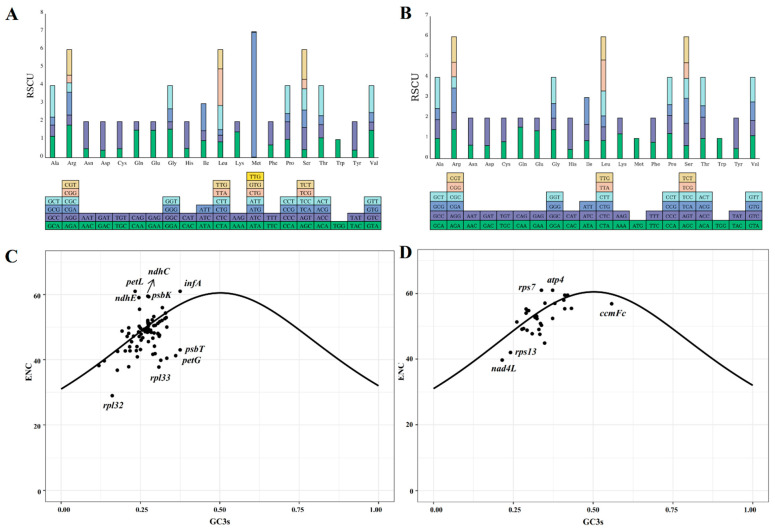
The relative synonymous codon usage values of each codon of chloroplast (**A**) and mitochondrial (**B**) genes. ENC-GC3s plots of chloroplast (**C**) and mitochondrial (**D**) genes of centipedegrass. The solid curve indicates the predicted gene positions when codon usage bias is determined exclusively by GC3s composition. (ENC: effective number of codons; GC3s: guanine+cytosine content at third synonymous codon positions).

The codon usage of 85 and 31 protein-coding genes (PCGs) from the chloroplast and mitochondrial genomes of centipedegrass, respectively, was examined. It was observed that a total of 32 codons in the chloroplast genome and 31 codons in the mitochondrial genome had relative synonymous codon usage (RSCU) values greater than one, signifying their frequent usage (Figure 2, Table S3). In particular, among the chloroplast PCGs, the codon AUG exhibited the highest RSCU value (6.94), followed by UUA (2.03) and AGA (1.81). For the mitochondrial PCGs, CAU demonstrated a notable RSCU value (1.55), followed by CAA (1.55) and GCU (1.53).

ENC-GC3s plots were generated for the chloroplast and mitochondrial PCGs (Figure 2C,D). The majority of the ENC values for PCGs in the organelle genomes of centipedegrass were found to be proximate to or above the ENC-GC3s curves, suggesting a pattern of codon usage that is consistent with neutral evolution. However, certain chloroplast genes (*rpl32*, *rpl33*, *petG*, *psbT*, and so on) and mitochondrial genes (*ccmFc*, *rpl13*, *nad4L*, and so on) exhibited ENC values that deviated significantly below the ENC-GC3s curves, indicating that these genes may be subject to substantial influence from natural selection.

### 3.3. Repeat Sequence Analysis

**Figure 3:**
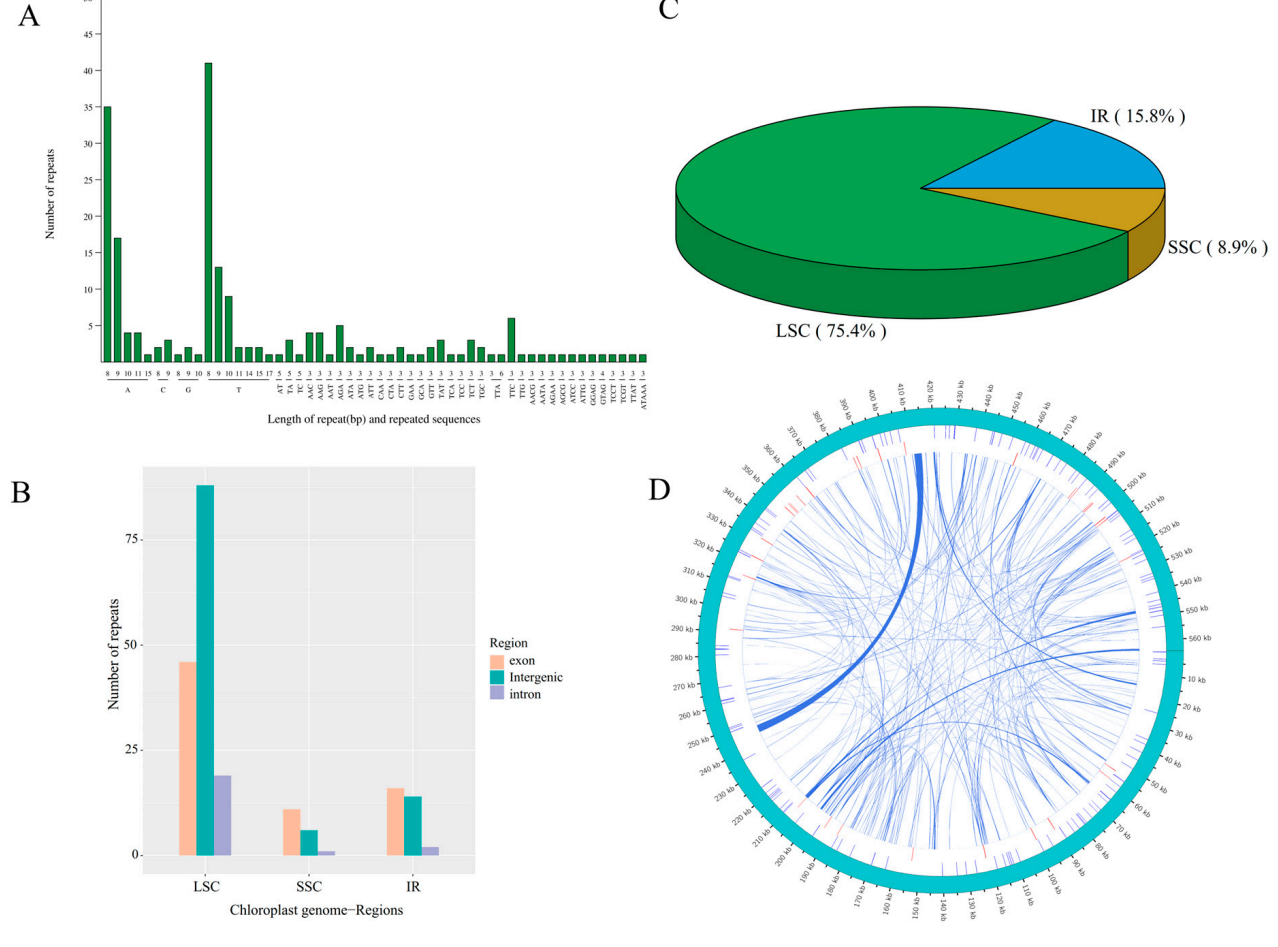
The type and number of simple sequence repeat (**A**), the number of repeats in each genomic region (exon, intergenic, and intron) in LSC, SSC, IR regions (**B**), the percentage of repeats in each LSC, SSC, IR regions (**C**) in chloroplast genome of centipedegrass, and a circus plot showing repeat sequences in the mitochondrial genome of centipedegrass (**D**). The outermost circle represents the mitochondrial genome sequence, followed inwardly by simple sequence repeats, tandem repeats, and dispersed repeats.

A total of 203 and 136 simple sequence repeats (SSRs) were identified in the chloroplast and mitochondrial genomes of centipedegrass, respectively, occupying 1806 bp (1.30% of the 139,107 bp) and 1606 bp (0.28% of the 564,432 bp) of the genome sequences. Predominantly, poly(A)/poly(T) SSRs were the most prevalent repeat types within both organelle genomes (Figure 3A). The large single-copy (LSC) region harbored the majority of these SSRs, particularly within its intergenic region (Figure 3B,C). In addition to SSRs, the mitochondrial genome of centipedegrass contained dispersed repeats, amounting to a total length of 23,850 bp (4.23% of the 564,432 bp), which comprised 114 forward and 138 palindromic repeats (Figure 3D, Table S4). These repeats were primarily located in intergenic regions or partial genic regions, including *nad7*, *atp4*, *cox2*, *rps3*, *ccmFc*, and *atp6*, among others.

### 3.4. Homology Sequences Between Chloroplast and Mitochondrial Genomes of Centipedegrass

**Figure 4:**
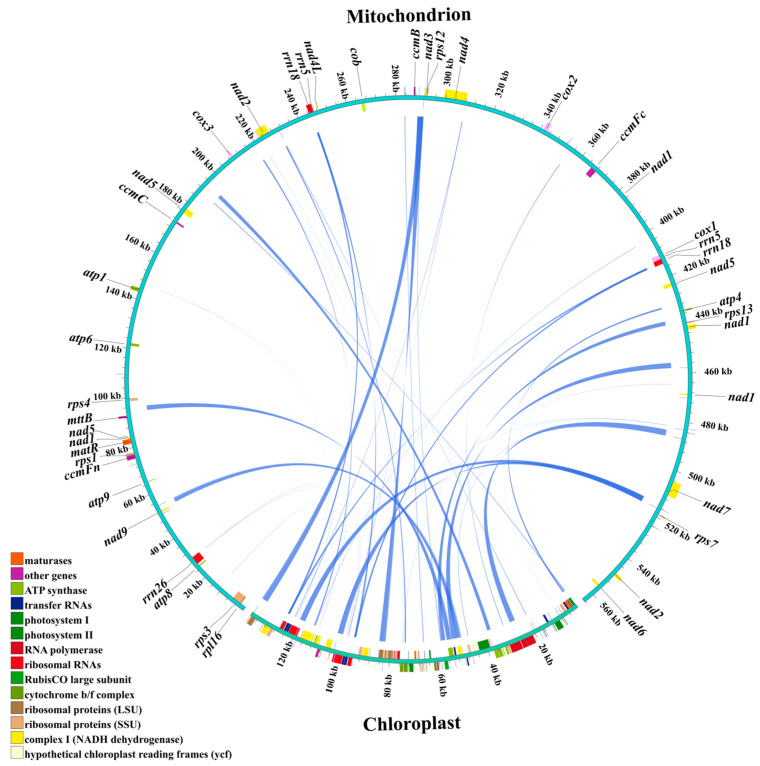
The homologous sequences between the chloroplast and mitochondrial genomes of centipedegrass. In the circular gene map, the transcription direction is indicated by arrow orientation: genes on the outer circle are transcribed clockwise, while inner circle genes are transcribed counterclockwise.

Homologous sequences between the chloroplast and mitochondrial genomes of centipedegrass were analyzed to investigate potential intracellular gene transfer (IGT) events (Figure 4). A total of 44 IGT events were identified, spanning a cumulative length of 30,355 bp, with 10 of these homologous sequences being greater than 1000 bp in length (Table S5). Notably, the complete genic regions of *rpl23*, *ndhC*, *atpE*, *atpB*, *rbcL*, and *ndhJ* in the chloroplast genome were found to have homologous counterparts in the mitochondrial genome. In contrast, the mitochondrial sequences that were homologous to the chloroplast genome were mainly located in intergenic regions or regions associated with tRNA/rRNA within the mitochondrial genome (Table S5).

### 3.5. Phylogenetic Analyses and Divergence Times in the Family Gramineae

**Figure 5:**
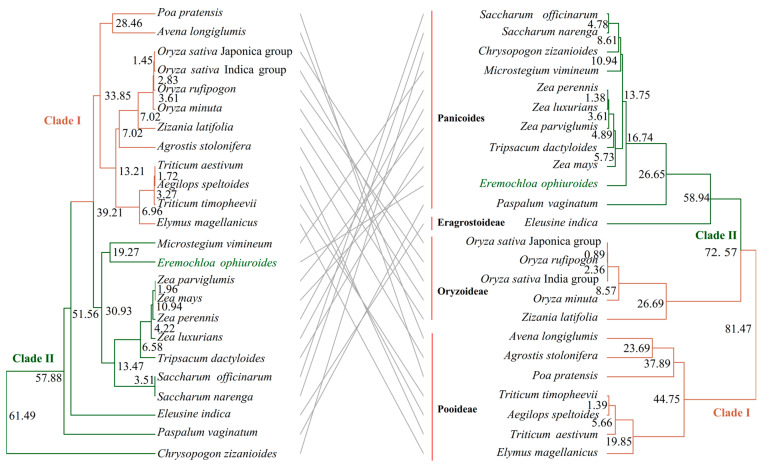
The tangled tree of Gramineae based on shared chloroplast (**right**) genes and mitochondrial (**left**) genes. The number in the node indicated the divergence time (Mya). The mitochondrial genome sequenced in this study was highlighted in green.

Given that the complete organelle genome does not contribute uniformly to phylogeographic or taxonomic studies, our focus was directed toward the functional genes that are conserved across all Gramineae species. We identified 2 and 41 common genes among the 24 Gramineae mitochondrial and chloroplast genomes, respectively (Table S6). Both ML trees could divide these species into two clades: Clade I and Clade II (Figure 5). The tangled tree showed high consistency between Clade I and Clade II. The chloroplast gene tree could effectively separate species from different subfamilies, indicating that the phylogenetic tree constructed by chloroplast genes was more consistent with the classical taxonomy than that of mitochondrial genes. Centipedegrass showed a closer relationship with *Microstegium ophiuroides* in the mitochondrial gene tree, with an estimated divergence time of 19.27 million years ago (Mya). However, in the chloroplast gene tree, centipedegrass diverged from the common ancestors of *Zea*, *Saccharum*, *Chrysopogon*, *Microstegium*, and *Tripsacum* genus at 13.75 Mya.

### 3.6. Structural Variations Among Mitochondrial Genomes

**Figure 6:**
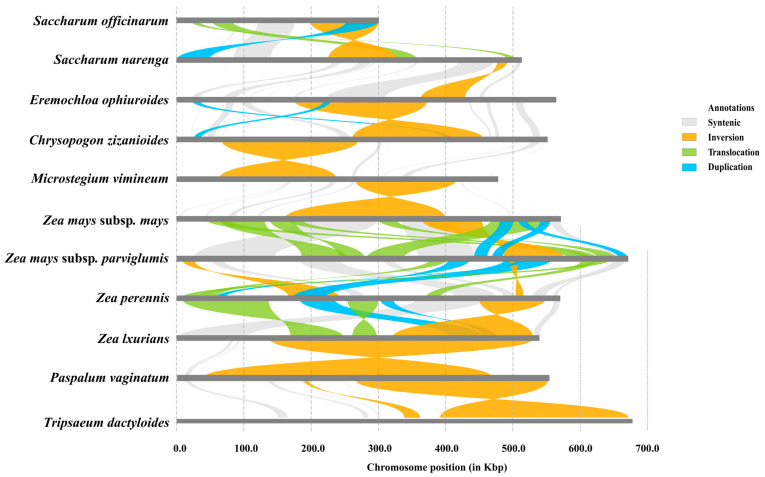
Pairwise structural variations between 11 mitochondrial genomes. Color-coded collinear relationships: gray; Syntenic blocks; Orange: Inversion events; Light green: Translocation events; Blue: Duplication events.

Given the prevalence of abundant structural variations (SVs) within plant mitochondrial genomes, we conducted pairwise alignments and variation detection among 11 Panicoides mitochondrial genomes (Figure 6). We detected a total of four SVs, including syntenic, inversion, translocation, and duplication, which underscore the occurrence of large-scale rearrangements within these mitochondrial genomes. Notably, one large syntenic region and a significant inversion variation were observed between the mitochondrial genomes of centipedegrass and *Saccharum narenga*, while one large inversion and two duplication events were identified between the mitochondrial genomes of centipedegrass and *Chrysopogon zizanioides*. The annotations revealed that these SVs impacted numerous genes (Table S7), highlighting the conservation of gene function despite the structural rearrangements for Gramineae mitochondrial genomes.

### 3.7. Genetic Diversity Indices Based on Organelle Genome Markers

A total of four organelle genome markers (*cox1*, *atp6*, *psbA-trnH*, and *rps-tRNAS*) were developed and used for DNA amplification across all wild centipedegrass accessions. However, due to the absence of sequence variation in the *rps-tRNAS* marker, only the remaining markers were used for the analysis of genetic diversity (Table 1). Among these, the mitochondrial gene cox1 exhibited the highest haplotype diversity (0.936), while *psbA-trnH* demonstrated the highest nucleotide diversity (0.01287). The Tajima’s D values calculated for the three markers were all less than zero, indicating a signature of population bottleneck or selection pressure.

Additionally, we constructed NJ trees for wild centipedegrass accessions based on the three markers (Figure S1). Notably, these three NJ trees failed to distinguish accessions from different geographical origins. However, an altitude-based clustering pattern emerged: four high-altitude accessions (E1, E11, E17, and E3) and four low-altitude accessions (E2, E7, E13, and E24) consistently formed separate monophyletic groups.

## 4. Discussions

### 4.1. Centipedegrass Mitogenome Reveals Evolutionary and Breeding Clues

The mitochondrial genome of plants is distinguished by its extensive genomic recombination and rearrangement, the gene transfer phenomena, and the prevalence of repetitive sequences. These characteristics lead to structural variability, with the genome existing as a single circular structure and complex combinations of circular and linear structures [37]. In the present study, we report that the mitochondrial genome of centipedegrass exhibited a circular structure, aligning with the pattern observed in other Poaceae species. To date, less than one-tenth as many plant mitochondrial genomes have been assembled compared to chloroplast genomes (http://www.ncbi.nlm.nih.gov/genome/organelle/, accessed on 20 May 2025) because of their high variability in size and structure, which makes them difficult to assemble. The newly assembled mitochondrial genome of centipedegrass represents the first one within the genus *Eremochloa*, which provides valuable data for phylogenetic studies at the genus level within the Poaceae family. The centipedegrass mitogenome enables precise lineage tracing in hybrid breeding, development of cytoplasmic male sterility (CMS)-specific molecular markers, and surveillance of cytoplasmic genome integrity to prevent unintended genetic introgression. For example, the gene *cox1* has emerged as a potential marker for distinguishing centipedegrass accessions, particularly those found at different altitudes. Previous research has suggested that *cox1* may be linked to adaptations to high-altitude environments [38], making it a promising tool for differentiation in future studies. This mitochondrial gene enables rapid screening of accessions across altitudinal gradients, substantially expediting the development of extremophile-resistant cultivars.

Phylogenetic analyses based on organelle genomes provided valuable insights into the evolutionary relationships and divergence times within the genus *Eremochloa*. Although complete mitochondrial and chloroplast genomes exhibit structural and evolutionary differences, the ML trees constructed based on mitochondrial and chloroplast genes shared across 24 Gramineae species consistently divided the species into two major clades (Figure 5), demonstrating high topological concordance. However, the chloroplast gene tree exhibited superior resolution in distinguishing species from different subfamilies, aligning more closely with classical taxonomic classifications compared to the mitochondrial gene tree. Notably, conflicting phylogenetic signals emerged between the two organelle genomes, particularly in the placement of centipedegrass, which clustered closely with *Microstegium ophiuroides* in the mitochondrial tree but showed an earlier divergence from a broader lineage, including *Zea*, *Saccharum*, and *Tripsacum*, in the chloroplast tree. These discrepancies may reflect differences in evolutionary rates, incomplete lineage sorting, or historical hybridization events, underscoring the complex evolutionary dynamics of organelle genomes [38]. While chloroplast genes provided stronger taxonomic resolution, mitochondrial genes offered complementary insights into divergence times and deep-node relationships [38]. Our findings highlight the importance of integrating data from both organelle genomes to reconstruct robust phylogenetic frameworks, particularly in resolving incongruences caused by differential inheritance patterns or heterogeneous substitution rates in the Gramineae family.

### 4.2. Repeated Sequences Identified in the Centipedegrass Mitogenome

Previous research has elucidated that intermolecular recombination driven by repetitive sequences and the acquisition of foreign DNA through horizontal or intracellular transfer are pivotal in shaping the mitochondrial genome and influencing its size [39,40]. In the present study, 25,456 bp (4.51% of the mitochondrial genome) of the repetitive sequences were identified in the centipedegrass mitochondrial genome. This percentage is lower than that observed in oat (233,082 bp, 42.5% of the mitochondrial genome) [41] and rice (51,328 bp, 9.97% of the mitochondrial genome) [42] and higher than in *Setaria italica* (13,056 bp, 2.92% of the mitochondrial genome) [43]. Notably, the length of the longest repeat sequence in the centipedegrass mitochondrial genome is the smallest (only 3292 bp) among these species, in contrast to the longest repeats in oat, rice, and *Setaria italica*, which are all greater than 6000 bp. Despite the influence of a multitude of factors on mitochondrial genome structure, it is hypothesized that the repeats of the centipedegrass mitochondrial genome might be a contributing factor in the centipedegrass mitochondrial genome, potentially explaining this unique genomic organization.

### 4.3. Plastid DNA Integration in Centipedegrass Mitogenome

Intracellular gene transfer (IGT), an event involving DNA exchange between organelles, has been recognized as a significant force driving mitochondrial evolution [44]. Plastid-derived DNA has been found to comprise a variable proportion of angiosperm mitochondrial DNAs, ranging from 0.1% to 10.3% of the total mitochondrial DNA content [45]. In this study, we identified a total of 44 IGT events and observed that plastid-derived sequences accounted for an intermediate proportion (5.38%) of the centipedegrass mitochondrial genome. While many chloroplast genes have been detected in mitochondrial genomes, such as the *atpI* gene in the mitochondrial genome of *Aeginetia indica* [46], our findings reveal that plastid-derived sequences in the centipedegrass mitochondrial genomes are primarily located in intergenic or tRNA/rRNA regions. These frequent IGT events do not directly impact mitochondrial PCGs but may influence mitochondrial function in centipedegrass by generating new gene variants, promoters, or incorporating new functional tRNA genes [47,48,49]. Environmental stress and the loss of mitochondrial membrane proteins may enhance the chloroplasts’ capacity to uptake exogenous DNA or facilitate the transfer of genes from the mitochondria [50,51]. Gene transfer hotspots, such as *rpl23* [52,53], *ndhC*/*ndhJ* [54], and *rbcL* [55], were also found to be homologous between the chloroplast and mitochondrial genomes of centipedegrass. The abundance of repeat sequences and the frequency of IGT events have collectively contributed to shaping the current mitochondrial genome structure and size of the centipedegrass mitochondrial genome. Future investigations should employ comparative transcriptomics to characterize expression patterns of these IGT regions, with particular emphasis on the *rpl23*, *ndhC*, and *rbcL* transfer hotspots. Functional validation through CRISPR-Cas9-mediated knockout studies and tRNA complementation assays will be essential to elucidate the mechanistic roles of IGT events in mitochondrial physiology and their evolutionary contributions to environmental adaptation.

### 4.4. Codon Usage Patterns in Centipedegrass Organelle Genomes

The variation in synonymous codons, i.e., codons that code for the same amino acid, is primarily reflected in the differences found in the third position of the codon [56]. In the current study, the PCGs from the organelle genomes of centipedegrass were found to frequently terminate with A/T in the third position of the codon, a pattern that aligns with the codon usage observed in numerous eukaryotes [57]. The ENC values are used to quantify the strength of codon usage bias. An ENC value lower than 35 is indicative of a strong codon preference [58]. Based on this criterion, all mitochondrial genes and chloroplast genes of centipedegrass, with the exception of *rpl32*, showed weak codon preference. This result suggests that most organelle-encoded proteins tolerate synonymous mutations to maintain genomic flexibility, while critical structural components like ribosomal proteins undergo purifying selection to preserve translational efficiency. Future studies should investigate whether this “selective modularity” pattern extends to other Poaceae species and if the rpl32 codon bias correlates with stress-responsive expression. Such findings would advance our understanding of how organelle genomes balance evolutionary flexibility with functional constraints.

## 5. Conclusions

This study presents the first mitochondrial genome of centipedegrass, revealing its circular structure with moderate repeats (4.51%) and plastid-derived sequences (5.38%). Phylogenetic analyses showed concordant yet conflicting signals between organelle genomes, suggesting complex evolutionary dynamics. The cox1 gene serves as an altitudinal adaptation marker, while the overall weak codon bias indicates genomic flexibility. These findings advance understanding of Eremochloa evolution and enable molecular marker development for breeding programs. Future work should explore the functional impacts of plastid-derived sequences on environmental adaptation.

## Figures and Tables

**Table 1:** The genetic diversity indices of three organelle genome markers.

Genes	Sample	Haplotype Number	Haplotype Diversity	Nucleotide Diversity	Tajima’s D
*cox1*	24	15	0.936	0.00305	−1.24332
*atp6*	24	2	0.359	0.00039	−0.66674
*psbA*-*trnH*	21	5	0.424	0.01287	−2.56233

## Data Availability

The sequencing raw data presented in the study are deposited in the China National GeneBank DataBase repository (https://db.cngb.org/), accession number CNP0006402.

## Supplementary Materials

The following supporting information can be downloaded at https://www.mdpi.com/article/10.3390/biology14080975/s1. Figure S1: The NJ trees for wild centipedegrass accessions using *cox1* (A), atp6 (B), and *psbA-trnH* (C) markers. Table S1: The primer sequences of four markers. Table S2: Basic information of experimental accessions. Table S3: The RSCU values of mitochondrial and chloroplast genes of centipedegrass. Table S4: The dispersed repeat sequences for the mitochondrial genome of centipedegrass. Table S5: The annotation results of the homologous sequences between the mitochondrial and chloroplast genomes of centipedegrass. Table S6: Common genes among the 24 Gramineae mitochondrial and chloroplast genomes. Table S7: The annotations for structural variations between centipedegrass and *Saccharum narenga*/*Chrysopogon zizanioides*.

## Abbreviations

The following abbreviations are used in this manuscript:

ENC	Effective number of codons
GC3s	3rd synonymous codon
IGT	Intracellular gene transfer
LSC	Large single copy
NGS	Next-generation sequencing
NJ	Neighbor-joining
PCGs	Protein-coding genes
RSCU	Relative synonymous codon usage
SSRs	Simple sequence repeats
SVs	Structural variations
