# Supplementary figures and images for: Organelle Genome Characteristics and Phylogenetic Analysis of a Warm-Season Turfgrass Eremochloa ophiuroides (Poaceae)
Source: Biology (Basel). 2025 Aug 1;14(8):975. doi: 10.3390/biology14080975 (PMC12384027; doi:10.3390/biology14080975)

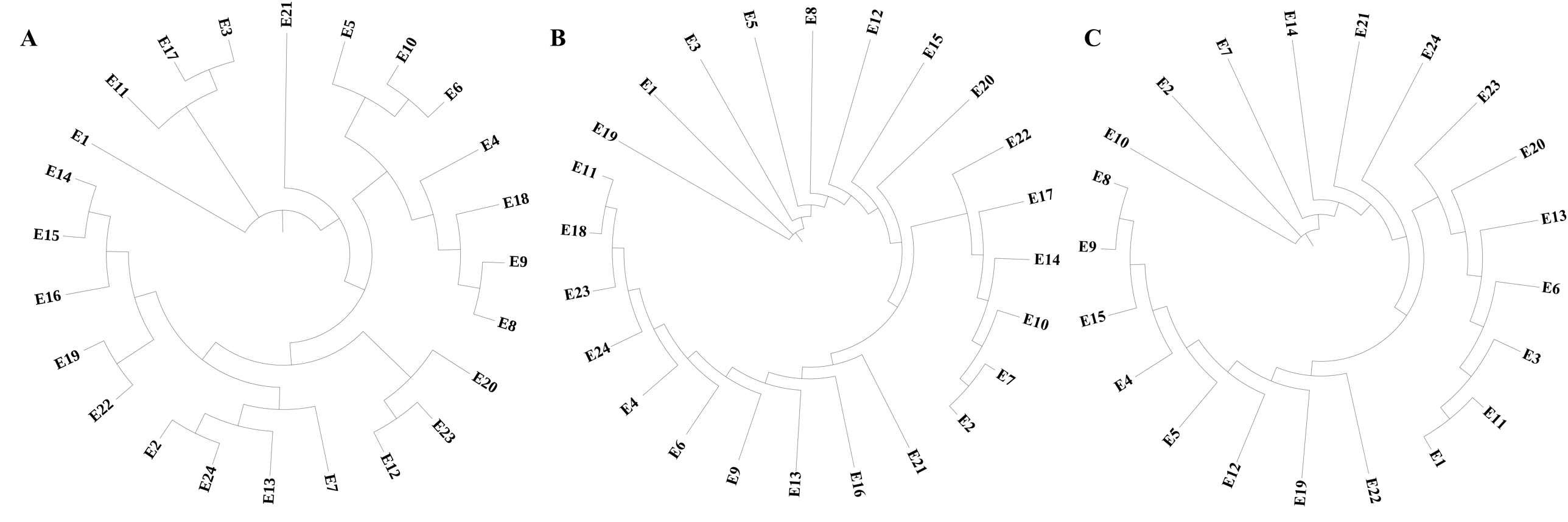

Supplement: Supplementary file 1 [file biology-14-00975-s001.zip › Fig S1.tif]
